# Supplementary material for: FBXL4 deficiency increases mitochondrial removal by autophagy
Source: EMBO Mol Med. 2020 Jun 11;12(7):e11659. doi: 10.15252/emmm.201911659 (PMC7338799; doi:10.15252/emmm.201911659)
Supplement: Supplementary file 1 — Appendix [file EMMM-12-e11659-s001.pdf]

# FBXL4 deficiency increases mitochondrial removal by autophagy

David Alsina, Oleksandr Lytovchenko, Aleksandra Schab, Ilian Atanassov, Florian Schober, Min Jiang, Camilla Koolmeister, Anna Wedell, Robert W. Taylor, Anna Wredenberg, Nils-Göran Larsson

Appendix document table of contents

- Appendix Table S1
- Appendix Table S2
- Appendix Table S3

**Appendix Table S1**. qPCR probe list used in the study

| Probe | Species | Reporter | Reference number |
|---|---|---|---|
| ACTB | Mus musculus | FAM | Mm01205647_g1 |
| B2M | Mus musculus | FAM | Mm00437762_m1 |
| FBXL4 | Mus musculus | FAM | Mm01217476_m1 |
| ND1 | Mus musculus | FAM | Mm04225274_s1 |
| ND3 | Mus musculus | FAM | Mm04225292_g1 |
| ND6 | Mus musculus | FAM | AIHSNT9 |
| CYTB | Mus musculus | FAM | Mm04225271_g1 |
| COX2 | Mus musculus | FAM | Mm03294838_g1 |
| ATP8 | Mus musculus | FAM | Mm04225236_g1 |
| TFAM | Mus musculus | FAM | Mm00447485_m1 |
| POLRMT | Mus musculus | FAM | Mm00553272_m1 |
| TFB2M | Mus musculus | FAM | Mm01620397_s1 |
| VDAC1 | Mus musculus | FAM | Mm00834272_m1 |
| ACTB | Homo Sapiens | FAM | Hs99999903_m1 |
| 18S | Homo Sapiens | FAM | Hs99999901_s1 |
| FBXL4 | Homo Sapiens | FAM | Hs01091896_m1 |
| ND1 | Homo Sapiens | FAM | Hs02596873_s1 |
| COX2 | Homo Sapiens | FAM | Hs02596865_g1 |
| ATP6 | Homo Sapiens | FAM | Hs02596862_g1 |
| RNR2 | Homo Sapiens | FAM | Hs02596860_s1 |
| VDAC1 | Homo Sapiens | FAM | Hs01631624_gH |
| HSP60 | Homo Sapiens | FAM | Hs01036753_g1 |
| TFAM | Homo Sapiens | FAM | Hs00273372_s1 |
| POLRMT | Homo Sapiens | FAM | Hs04187596_g1 |
| TFB2M | Homo Sapiens | FAM | Hs00915025_m1 |
| GAPDH | Homo Sapiens | FAM | Hs03929097_g1 |
| LAMP1 | Homo Sapiens | FAM | Hs00931461_m1 |

**Appendix Table S2**. Primary antibody list used in the study

| Antigen | Source | Company | Catalog Number | Method | Dilution |
|---|---|---|---|---|---|
| Hsp60 | Mouse | Enzo Lifesciences | AB1-SPA-807-E | WB | 1:1000 |
| Total OXPHOS Rodent | Mouse | Abcam | MS604-300 | WB | 1:1000 |
| Tfam | Rabbit | Abcam | ab-131607 | WB | 1:1000 |
| ActB | mouse | Abcam | ab8226 | WB | 1:1000 |
| Vdac1 | mouse | abcam | ab128568 | WB | 1:1000 |
| Uqcrc2 | Mouse | Abcam | ab14745 | WB | 1:1000 |
| Atp5a | Mouse | Abcam | ab14748 | WB | 1:1000 |
| Sdha | Mouse | Abcam | ab14715 | WB | 1:1000 |
| Gapdh | Mouse | Abcam | ab8245 | WB | 1:1000 |
| Mtco2 | Mouse | Abcam | ab110258 | WB | 1:1000 |
| Lamp2 | mouse | abcam | ab25631 | IF | 1:250 |
| Tom20 | Rabbit | Cell Signaling | 13929S | WB | 1:1000 |
| Lamp1 | rabbit | Cell Signalling | 9091S | WB | 1:1000 |
| Rpl6 | mouse | Abcam | ab126100 | WB | 1:1000 |
| Mrpl28 | Rabbit | Abcam | ab196842 | WB | 1:1000 |
| Lc3II | rabbit | Novus Bio | 100-2331 | WB | 1:1000 |
| p62/Sqstm1 | mouse | Abcam | ab56416 | WB | 1:1000 |
| Ub | mouse | Cell Signalling | 3936S | WB | 1:500 |
| Iba1 | Rabbit | Wako | 0919-19741 | IHC | 1:1000 |

**Appendix Table S3**. Statistics summary table

| Figure panel | Statistical test | Comparison | n | p-value | Summary |
|---|---|---|---|---|---|
| 1B | Chi2 test | Embryo vs Expected Mendelian distribution (%) | 79 | 0,15 | n.s |
| | Chi2 test | Mice vs Expected Mendelian distribution(%) | 339 | 1,3408E-09 | *** |
| | | | | | |
| 1D | Student's t-test | Fbxl4+/+ vs Fbxl4 -/- | 5 | 0,0373 | * |
| | | | | | |
| 1F | Student's t-test | Male Fbxl4 $^{+/+}$ vs Fbxl4 $^{-/-}$ | 5 | 2,96858E-06 | *** |
| | Student's t-test | Female Fbxl4 $^{+/+}$ vs Fbxl4 $^{-/-}$ | 6 (wt), 11 (KO) | 0,0024 | ** |
| | | | | | |
| 1G | Student's t-test | Male Fbxl4 $^{+/+}$ vs Fbxl4 $^{-/-}$ | 5 | 0,0678 | n.s |
| | Student's t-test | Female Fbxl4 $^{+/+}$ vs Fbxl4 $^{-/-}$ | 5 (wt), 11 (KO) | 0,8276 | n.s |
| | | | | | |
| 1H | Student's t-test | Male Fbxl4 $^{+/+}$ vs Fbxl4 $^{-/-}$ | 4 (wt), 5 (KO) | 0,0389 | * |
| | Student's t-test | Female Fbxl4 $^{+/+}$ vs Fbxl4 $^{-/-}$ | 5 (wt), 11 (KO) | 0,3619 | n.s |
| | | | | | |
| 1I | Student's t-test | Male Fbxl4 $^{+/+}$ vs Fbxl4 $^{-/-}$ | 4 (wt), 5 (KO) | 0,0412 | * |
| | Student's t-test | Female Fbxl4 $^{+/+}$ vs Fbxl4 $^{-/-}$ | 5 (wt), 11 (KO) | 0,1842 | n.s |
| | | | | | |
| 2A | Student's t-test | Heart, Fbxl4 $^{+/+}$ vs Fbxl4 $^{-/-}$ | wt (6), KO (8) | 0.3242 | n.s |
| | Student's t-test | Liver, Fbxl4 $^{+/+}$ vs Fbxl4 $^{-/-}$ | wt (6), KO (8) | 0.000002 | *** |
| | Student's t-test | Kidney, Fbxl4 $^{+/+}$ vs Fbxl4 $^{-/-}$ | wt (6), KO (8) | 0.00256 | ** |
| | Student's t-test | Brain, Fbxl4 $^{+/+}$ vs Fbxl4 $^{-/-}$ | wt (6), KO (8) | 0.001496 | ** |
| | Student's t-test | Quadrieps, Fbxl4 $^{+/+}$ vs Fbxl4 $^{-/-}$ | wt (6), KO (8) | 0.5574 | n.s |
| | | | | | |
| 2B | Student's t-test | Heart, ND3, Fbxl4 $^{+/+}$ vs Fbxl4 $^{-/-}$ | wt (6), KO (7) | 0,00107 | ** |
| | Student's t-test | Heart, ND6, Fbxl4 $^{+/+}$ vs Fbxl4 $^{-/-}$ | wt (6), KO (7) | 0,00663 | ** |
| | Student's t-test | Heart, CYTB, Fbxl4 $^{+/+}$ vs Fbxl4 $^{-/-}$ | wt (6), KO (7) | 0,00082 | *** |
| | Student's t-test | Heart, COX2, Fbxl4 $^{+/+}$ vs Fbxl4 $^{-/-}$ | wt (6), KO (7) | 0,03610 | * |
| | Student's t-test | Heart, ATP8, Fbxl4 $^{+/+}$ vs Fbxl4 $^{-/-}$ | wt (6), KO (7) | 0,00178 | ** |
| | Student's t-test | Heart, TFAM, Fbxl4 $^{+/+}$ vs Fbxl4 $^{-/-}$ | wt (6), KO (7) | 0,68137 | n.s |
| | Student's t-test | Heart, POLRMT, Fbxl4 $^{+/+}$ vs Fbxl4 $^{-/-}$ | wt (6), KO (7) | 0,74295 | n.s |
| | Student's t-test | Heart, TFB2M, Fbxl4 $^{+/+}$ vs Fbxl4 $^{-/-}$ | wt (6), KO (7) | 0,07989 | n.s |
| | Student's t-test | Heart, VDAC, Fbxl4 $^{+/+}$ vs Fbxl4 $^{-/-}$ | wt (6), KO (7) | 0,53017 | n.s |
| | Student's t-test | Heart, FBXL4, Fbxl4 $^{+/+}$ vs Fbxl4 $^{-/-}$ | wt (6), KO (7) | 0,01407 | * |
| | Student's t-test | Liver, ND3, Fbxl4 $^{+/+}$ vs Fbxl4 $^{-/-}$ | wt (5), KO (8) | 0,00650 | ** |
| | Student's t-test | Liver, ND6, Fbxl4 $^{+/+}$ vs Fbxl4 $^{-/-}$ | wt (5), KO (8) | 0,00118 | ** |
| | Student's t-test | Liver, CYTB, Fbxl4 $^{+/+}$ vs Fbxl4 $^{-/-}$ | wt (5), KO (8) | 0,00019 | *** |
| | Student's t-test | Liver, COX2, Fbxl4 $^{+/+}$ vs Fbxl4 $^{-/-}$ | wt (5), KO (8) | 0,00542 | ** |
| | Student's t-test | Liver, ATP8, Fbxl4 $^{+/+}$ vs Fbxl4 $^{-/-}$ | wt (5), KO (8) | 0,00008 | *** |
| | Student's t-test | Liver, TFAM, Fbxl4 $^{+/+}$ vs Fbxl4 $^{-/-}$ | wt (5), KO (8) | 0,69154 | n.s |
| | Student's t-test | Liver, POLRMT, Fbxl4 $^{+/+}$ vs Fbxl4 $^{-/-}$ | wt (5), KO (8) | 0,47252 | n.s |
| | Student's t-test | Liver, TFB2M, Fbxl4 $^{+/+}$ vs Fbxl4 $^{-/-}$ | wt (5), KO (8) | 0,55530 | n.s |
| | Student's t-test | Liver, VDAC, Fbxl4 $^{+/+}$ vs Fbxl4 $^{-/-}$ | wt (5), KO (8) | 0,92194 | n.s |
| | Student's t-test | Liver, FBXL4, Fbxl4 $^{+/+}$ vs Fbxl4 $^{-/-}$ | wt (5), KO (8) | 0,00188 | ** |
| | Student's t-test | Kidney, ND3, Fbxl4 $^{+/+}$ vs Fbxl4 $^{-/-}$ | wt (6), KO (8) | 0,06788 | n.s |
| | Student's t-test | Kidney, ND6, Fbxl4 $^{+/+}$ vs Fbxl4 $^{-/-}$ | wt (6), KO (8) | 0,12486 | n.s |
| | Student's t-test | Kidney, CYTB, Fbxl4 $^{+/+}$ vs Fbxl4 $^{-/-}$ | wt (6), KO (8) | 0,00891 | ** |
| | Student's t-test | Kidney, COX2, Fbxl4 $^{+/+}$ vs Fbxl4 $^{-/-}$ | wt (6), KO (8) | 0,00072 | *** |
| | Student's t-test | Kidney, ATP8, Fbxl4 $^{+/+}$ vs Fbxl4 $^{-/-}$ | wt (6), KO (8) | 0,00002 | *** |
| | Student's t-test | Kidney, TFAM, Fbxl4 $^{+/+}$ vs Fbxl4 $^{-/-}$ | wt (6), KO (8) | 0,34896 | n.s |
| | Student's t-test | Kidney, POLRMT, Fbxl4 $^{+/+}$ vs Fbxl4 $^{-/-}$ | wt (6), KO (8) | 0,91836 | n.s |
| | Student's t-test | Kidney, TFB2M, Fbxl4 $^{+/+}$ vs Fbxl4 $^{-/-}$ | wt (6), KO (8) | 0,54576 | n.s |
| | Student's t-test | Kidney, VDAC, Fbxl4 $^{+/+}$ vs Fbxl4 $^{-/-}$ | wt (6), KO (8) | 0,50744 | n.s |
| | Student's t-test | Kidney, FBXL4, Fbxl4 $^{+/+}$ vs Fbxl4 $^{-/-}$ | wt (6), KO (8) | 1,47E-08 | *** |
| | Student's t-test | Brain, ND3, Fbxl4 $^{+/+}$ vs Fbxl4 $^{-/-}$ | wt (6), KO (8) | 0,03641 | * |
| | Student's t-test | Brain, ND6, Fbxl4 $^{+/+}$ vs Fbxl4 $^{-/-}$ | wt (6), KO (8) | 0,00565 | ** |
| | Student's t-test | Brain, CYTB, Fbxl4 $^{+/+}$ vs Fbxl4 $^{-/-}$ | wt (6), KO (8) | 0,01696 | * |
| | Student's t-test | Brain, COX2, Fbxl4 $^{+/+}$ vs Fbxl4 $^{-/-}$ | wt (6), KO (8) | 0,09564 | n.s |
| | Student's t-test | Brain, ATP8, Fbxl4 $^{+/+}$ vs Fbxl4 $^{-/-}$ | wt (6), KO (8) | 0,02101 | * |
| | Student's t-test | Brain, TFAM, Fbxl4 $^{+/+}$ vs Fbxl4 $^{-/-}$ | wt (6), KO (8) | 0,57586 | n.s |
| | Student's t-test | Brain, POLRMT, Fbxl4 $^{+/+}$ vs Fbxl4 $^{-/-}$ | wt (6), KO (8) | 0,99567 | n.s |
| | Student's t-test | Brain, TFB2M, Fbxl4 $^{+/+}$ vs Fbxl4 $^{-/-}$ | wt (6), KO (8) | 0,66057 | n.s |
| | Student's t-test | Brain, VDAC, Fbxl4 $^{+/+}$ vs Fbxl4 $^{-/-}$ | wt (6), KO (8) | 0,86484 | n.s |
| | Student's t-test | Brain, FBXL4, Fbxl4 $^{+/+}$ vs Fbxl4 $^{-/-}$ | wt (6), KO (8) | 8,18E-05 | *** |
| | | | | | |
| 2D | Student's t-test | HSP60, Fbxl4 $^{+/+}$ vs Fbxl4 $^{-/-}$ | 4 | 0,00823 | ** |
| | Student's t-test | ATP5A, Fbxl4 $^{+/+}$ vs Fbxl4 $^{-/-}$ | 4 | 0,00604 | ** |
| | Student's t-test | UQCRC2, Fbxl4 $^{+/+}$ vs Fbxl4 $^{-/-}$ | 4 | 0,000001 | *** |
| | Student's t-test | MTCO1, Fbxl4 $^{+/+}$ vs Fbxl4 $^{-/-}$ | 4 | 0,00207 | ** |
| | Student's t-test | SDHB, Fbxl4 $^{+/+}$ vs Fbxl4 $^{-/-}$ | 4 | 0,00104 | ** |
| | Student's t-test | NDUFB8, Fbxl4 $^{+/+}$ vs Fbxl4 $^{-/-}$ | 4 | 0,07435 | n.s |
| | Student's t-test | TFAM, Fbxl4 $^{+/+}$ vs Fbxl4 $^{-/-}$ | 4 | 0,07048 | n.s |
| | Student's t-test | ACTB, Fbxl4 $^{+/+}$ vs Fbxl4 $^{-/-}$ | 4 | 0,17800 | n.s |

| Figure panel | Statistical test | Comparison | n | p-value | Summary |
|---|---|---|---|---|---|
| 3B | Student's t-test | Controls vs Patients | 3 | | n.s |
| | | | | | |
| 3D | Student's t-test | VDAC1, Controls vs Patients | 3 | 0,00068 | *** |
| | Student's t-test | HSP60, Controls vs Patients | 3 | 0,03502 | * |
| | Student's t-test | UQCRC2, Controls vs Patients | 3 | 0,00519 | ** |
| | Student's t-test | MTCO2, Controls vs Patients | 3 | 0,01441 | * |
| | Student's t-test | SDHA, Controls vs Patients | 3 | 0,03171 | * |
| | Student's t-test | ATP5A, Controls vs Patients | 3 | 0,01299 | * |
| | Student's t-test | MRPL28, Controls vs Patients | 3 | 0,05439 | n.s |
| | Student's t-test | TFAM, Controls vs Patients | 3 | 0,01586 | * |
| | Student's t-test | TOM20, Controls vs Patients | 3 | 0,02768 | * |
| | Student's t-test | GAPDH, Controls vs Patients | 3 | 0,34086 | n.s |
| | Student's t-test | RPL6, Controls vs Patients | 3 | 0,88309 | n.s |
| | Student's t-test | ACTB, Controls vs Patients | 3 | 0,98986 | n.s |
| | Student's t-test | LAMP1, Controls vs Patients | 3 | 0,09369 | n.s |
| | | | | | |
| 3E | Student's t-test | FBXL4, Controls vs Patients | 3 | 0,14880 | n.s |
| | Student's t-test | ND1, Controls vs Patients | 3 | 0,10418 | n.s |
| | Student's t-test | MTCO2, Controls vs Patients | 3 | 0,06840 | n.s |
| | Student's t-test | ATP6, Controls vs Patients | 3 | 0,07822 | n.s |
| | Student's t-test | RNR2, Controls vs Patients | 3 | 0,10942 | n.s |
| | Student's t-test | VDAC1, Controls vs Patients | 3 | 0,72615 | n.s |
| | Student's t-test | HSP60, Controls vs Patients | 3 | 0,41906 | n.s |
| | Student's t-test | TFAM, Controls vs Patients | 3 | 0,90184 | n.s |
| | Student's t-test | POLRMT, Controls vs Patients | 3 | 0,79077 | n.s |
| | Student's t-test | TFB2M, Controls vs Patients | 3 | 0,50759 | n.s |
| | Student's t-test | GAPDH, Controls vs Patients | 3 | 0,80683 | n.s |
| | Student's t-test | LAMP1, Controls vs Patients | 3 | 0,81890 | n.s |
| | | | | | |
| 4C | Student's t-test | Pulse, wt vs KO | 4 | 0,263117 | n.s |
| | Student's t-test | 24h Chase, wt vs KO | 4 | 0,0016 | * |
| | | | | | |
| 4D | Student's t-test | 24h Chase, wt vs KO | 4 | 0,01297 | * |
| | Student's t-test | 24h Chase + Epoxo, wt vs KO | 3 | 0,03528 | * |
| | Student's t-test | 24h Chase + $NH_4Cl$, wt vs KO | 4 | 0,51949 | n.s |
| | | | | | |
| 5B | Non parametric Mann-Whitney | Control vs Patients | Control (67), Patients (303) | 0,0006 | *** |
| | | | | | |
| EV1A | Student's t-test | Male Fbxl4 $^{+/+}$ vs Fbxl4 $^{-/-}$ | Wt (4), KO (5) | 0,000031 | *** |
| | Student's t-test | Female Fbxl4 $^{+/+}$ vs Fbxl4 $^{-/-}$ | 5 (wt), 11 (KO) | 0,000045 | *** |
| | | | | | |
| EV1B | Student's t-test | Male Fbxl4 $^{+/+}$ vs Fbxl4 $^{-/-}$ | Wt (5), KO (5) | 0,0000016 | *** |
| | Student's t-test | Female Fbxl4 $^{+/+}$ vs Fbxl4 $^{-/-}$ | 5 (wt), 11 (KO) | 0,000213 | *** |
| | | | | | |
| EV1C | Student's t-test | Male Fbxl4 $^{+/+}$ vs Fbxl4 $^{-/-}$ | Wt (4), KO (5) | 0,11906 | n.s |
| | Student's t-test | Female Fbxl4 $^{+/+}$ vs Fbxl4 $^{-/-}$ | 5 (wt), 11 (KO) | 0,00360 | ** |
| | | | | | |
| EV2I | Student's t-test | CI, Fbxl4 $^{+/+}$ vs Fbxl4 $^{-/-}$ | 5 | 0,82240 | n.s |
| | Student's t-test | CI+III, Fbxl4 $^{+/+}$ vs Fbxl4 $^{-/-}$ | 5 | 0,47383 | n.s |
| | Student's t-test | CII, Fbxl4 $^{+/+}$ vs Fbxl4 $^{-/-}$ | 5 | 0,51135 | n.s |
| | Student's t-test | CII+III, Fbxl4 $^{+/+}$ vs Fbxl4 $^{-/-}$ | 5 | 0,65070 | n.s |
| | Student's t-test | CIV, Fbxl4 $^{+/+}$ vs Fbxl4 $^{-/-}$ | 5 | 0,00906 | ** |
| | | | | | |
| EV3B | Student's t-test | Controls vs Patients | 3 | 0,7305 | n.s |

| Figure panel | Statistical test | Comparison | n | p-value | Summary |
|---|---|---|---|---|---|
| EV4C | One-way ANOVA - Tukey's post hoc | Control vs. Patients | 12 (3 Control/Patient x 4 technical replicates) | >0,9999 | ns |
| | One-way ANOVA - Tukey's post hoc | Control vs. Control NH4 | 12 (3 Control/Patient x 4 technical replicates) | 0,5671 | ns |
| | One-way ANOVA - Tukey's post hoc | Control vs. Patients NH4 | 12 (3 Control/Patient x 4 technical replicates) | 0,4044 | ns |
| | One-way ANOVA - Tukey's post hoc | Control vs. Control - FBS | 12 (3 Control/Patient x 4 technical replicates) | 0,9995 | ns |
| | One-way ANOVA - Tukey's post hoc | Control vs. Patients - FBS | 12 (3 Control/Patient x 4 technical replicates) | 0,9994 | ns |
| | One-way ANOVA - Tukey's post hoc | Control vs. Control NH4 - FBS | 12 (3 Control/Patient x 4 technical replicates) | 0,0333 | * |
| | One-way ANOVA - Tukey's post hoc | Control vs. Patients NH4 - FBS | 12 (3 Control/Patient x 4 technical replicates) | 0,0001 | *** |
| | One-way ANOVA - Tukey's post hoc | Patients vs. Control NH4 | 12 (3 Control/Patient x 4 technical replicates) | 0,5901 | ns |
| | One-way ANOVA - Tukey's post hoc | Patients vs. Patients NH4 | 12 (3 Control/Patient x 4 technical replicates) | 0,4257 | ns |
| | One-way ANOVA - Tukey's post hoc | Patients vs. Control - FBS | 12 (3 Control/Patient x 4 technical replicates) | 0,9997 | ns |
| | One-way ANOVA - Tukey's post hoc | Patients vs. Patients - FBS | 12 (3 Control/Patient x 4 technical replicates) | 0,9996 | ns |
| | One-way ANOVA - Tukey's post hoc | Patients vs. Control NH4 - FBS | 12 (3 Control/Patient x 4 technical replicates) | 0,0367 | * |
| | One-way ANOVA - Tukey's post hoc | Patients vs. Patients NH4 - FBS | 12 (3 Control/Patient x 4 technical replicates) | 0,0002 | *** |
| | One-way ANOVA - Tukey's post hoc | Control NH4 vs. Patients NH4 | 12 (3 Control/Patient x 4 technical replicates) | >0,9999 | ns |
| | One-way ANOVA - Tukey's post hoc | Control NH4 vs. Control - FBS | 12 (3 Control/Patient x 4 technical replicates) | 0,8743 | ns |
| | One-way ANOVA - Tukey's post hoc | Control NH4 vs. Patients - FBS | 12 (3 Control/Patient x 4 technical replicates) | 0,88 | ns |
| | One-way ANOVA - Tukey's post hoc | Control NH4 vs. Control NH4 - FBS | 12 (3 Control/Patient x 4 technical replicates) | 0,868 | ns |
| | One-way ANOVA - Tukey's post hoc | Control NH4 vs. Patients NH4 - FBS | 12 (3 Control/Patient x 4 technical replicates) | 0,0734 | ns |
| | One-way ANOVA - Tukey's post hoc | Patients NH4 vs. Control - FBS | 12 (3 Control/Patient x 4 technical replicates) | 0,7464 | ns |
| | One-way ANOVA - Tukey's post hoc | Patients NH4 vs. Patients - FBS | 12 (3 Control/Patient x 4 technical replicates) | 0,7546 | ns |
| | One-way ANOVA - Tukey's post hoc | Patients NH4 vs. Control NH4 - FBS | 12 (3 Control/Patient x 4 technical replicates) | 0,9503 | ns |
| | One-way ANOVA - Tukey's post hoc | Patients NH4 vs. Patients NH4 - FBS | 12 (3 Control/Patient x 4 technical replicates) | 0,1334 | ns |
| | One-way ANOVA - Tukey's post hoc | Control - FBS vs. Patients - FBS | 12 (3 Control/Patient x 4 technical replicates) | >0,9999 | ns |
| | One-way ANOVA - Tukey's post hoc | Control - FBS vs. Control NH4 - FBS | 12 (3 Control/Patient x 4 technical replicates) | 0,1284 | ns |
| | One-way ANOVA - Tukey's post hoc | Control - FBS vs. Patients NH4 - FBS | 12 (3 Control/Patient x 4 technical replicates) | 0,0011 | ** |
| | One-way ANOVA - Tukey's post hoc | Patients - FBS vs. Control NH4 - FBS | 12 (3 Control/Patient x 4 technical replicates) | 0,1325 | ns |
| | One-way ANOVA - Tukey's post hoc | Patients - FBS vs. Patients NH4 - FBS | 12 (3 Control/Patient x 4 technical replicates) | 0,0011 | ** |
| | One-way ANOVA - Tukey's post hoc | Control NH4 - FBS vs. Patients NH4 - FB | 12 (3 Control/Patient x 4 technical replicates) | 0,7562 | ns |
| | | | | | |
| EV4D | One-way ANOVA - Tukey's post hoc | Control vs. Patients | 12 (3 Control/Patient x 4 technical replicates) | >0,9999 | ns |
| | One-way ANOVA - Tukey's post hoc | Control vs. Control NH4 | 12 (3 Control/Patient x 4 technical replicates) | 0,8886 | ns |
| | One-way ANOVA - Tukey's post hoc | Control vs. Patients NH4 | 12 (3 Control/Patient x 4 technical replicates) | 0,9876 | ns |
| | One-way ANOVA - Tukey's post hoc | Control vs. Control - FBS | 12 (3 Control/Patient x 4 technical replicates) | 0,983 | ns |
| | One-way ANOVA - Tukey's post hoc | Control vs. Patients - FBS | 12 (3 Control/Patient x 4 technical replicates) | >0,9999 | ns |
| | One-way ANOVA - Tukey's post hoc | Control vs. Control NH4 - FBS | 12 (3 Control/Patient x 4 technical replicates) | >0,9999 | ns |
| | One-way ANOVA - Tukey's post hoc | Control vs. Patients NH4 - FBS | 12 (3 Control/Patient x 4 technical replicates) | 0,9779 | ns |
| | One-way ANOVA - Tukey's post hoc | Patients vs. Control NH4 | 12 (3 Control/Patient x 4 technical replicates) | 0,9454 | ns |
| | One-way ANOVA - Tukey's post hoc | Patients vs. Patients NH4 | 12 (3 Control/Patient x 4 technical replicates) | 0,9645 | ns |
| | One-way ANOVA - Tukey's post hoc | Patients vs. Control - FBS | 12 (3 Control/Patient x 4 technical replicates) | 0,9548 | ns |
| | One-way ANOVA - Tukey's post hoc | Patients vs. Patients - FBS | 12 (3 Control/Patient x 4 technical replicates) | >0,9999 | ns |
| | One-way ANOVA - Tukey's post hoc | Patients vs. Control NH4 - FBS | 12 (3 Control/Patient x 4 technical replicates) | >0,9999 | ns |
| | One-way ANOVA - Tukey's post hoc | Patients vs. Patients NH4 - FBS | 12 (3 Control/Patient x 4 technical replicates) | 0,9933 | ns |
| | One-way ANOVA - Tukey's post hoc | Control NH4 vs. Patients NH4 | 12 (3 Control/Patient x 4 technical replicates) | 0,3644 | ns |
| | One-way ANOVA - Tukey's post hoc | Control NH4 vs. Control - FBS | 12 (3 Control/Patient x 4 technical replicates) | 0,3364 | ns |
| | One-way ANOVA - Tukey's post hoc | Control NH4 vs. Patients - FBS | 12 (3 Control/Patient x 4 technical replicates) | 0,8261 | ns |
| | One-way ANOVA - Tukey's post hoc | Control NH4 vs. Control NH4 - FBS | 12 (3 Control/Patient x 4 technical replicates) | 0,8261 | ns |
| | One-way ANOVA - Tukey's post hoc | Control NH4 vs. Patients NH4 - FBS | 12 (3 Control/Patient x 4 technical replicates) | >0,9999 | ns |
| | One-way ANOVA - Tukey's post hoc | Patients NH4 vs. Control - FBS | 12 (3 Control/Patient x 4 technical replicates) | >0,9999 | ns |
| | One-way ANOVA - Tukey's post hoc | Patients NH4 vs. Patients - FBS | 12 (3 Control/Patient x 4 technical replicates) | 0,9956 | ns |
| | One-way ANOVA - Tukey's post hoc | Patients NH4 vs. Control NH4 - FBS | 12 (3 Control/Patient x 4 technical replicates) | 0,9956 | ns |
| | One-way ANOVA - Tukey's post hoc | Patients NH4 vs. Patients NH4 - FBS | 12 (3 Control/Patient x 4 technical replicates) | 0,5939 | ns |
| | One-way ANOVA - Tukey's post hoc | Control - FBS vs. Patients - FBS | 12 (3 Control/Patient x 4 technical replicates) | 0,9935 | ns |
| | One-way ANOVA - Tukey's post hoc | Control - FBS vs. Control NH4 - FBS | 12 (3 Control/Patient x 4 technical replicates) | 0,9935 | ns |
| | One-way ANOVA - Tukey's post hoc | Control - FBS vs. Patients NH4 - FBS | 12 (3 Control/Patient x 4 technical replicates) | 0,5614 | ns |
| | One-way ANOVA - Tukey's post hoc | Patients - FBS vs. Control NH4 - FBS | 12 (3 Control/Patient x 4 technical replicates) | >0,9999 | ns |
| | One-way ANOVA - Tukey's post hoc | Patients - FBS vs. Patients NH4 - FBS | 12 (3 Control/Patient x 4 technical replicates) | 0,9539 | ns |
| | One-way ANOVA - Tukey's post hoc | Control NH4 - FBS vs. Patients NH4 - FB | 12 (3 Control/Patient x 4 technical replicates) | 0,9539 | ns |
| | | | | | |
| EV4F | Student's t-test | Ubiquitin, Controls vs Patients | 3 | 0,580488 | ns |
| | Student's t-test | p62, Controls vs Patients | 3 | 0,745301 | ns |
| | Student's t-test | LC3-II, Controls vs Patients | 3 | 0,244891 | ns |
