# Supplementary material for: FBXL4 deficiency increases mitochondrial removal by autophagy
Source: EMBO Mol Med. 2020 Jun 11;12(7):e11659. doi: 10.15252/emmm.201911659 (PMC7338799; doi:10.15252/emmm.201911659)
Supplement: Supplementary file 2 — Expanded View Figures PDF [file EMMM-12-e11659-s002.pdf]

Expanded View Figures

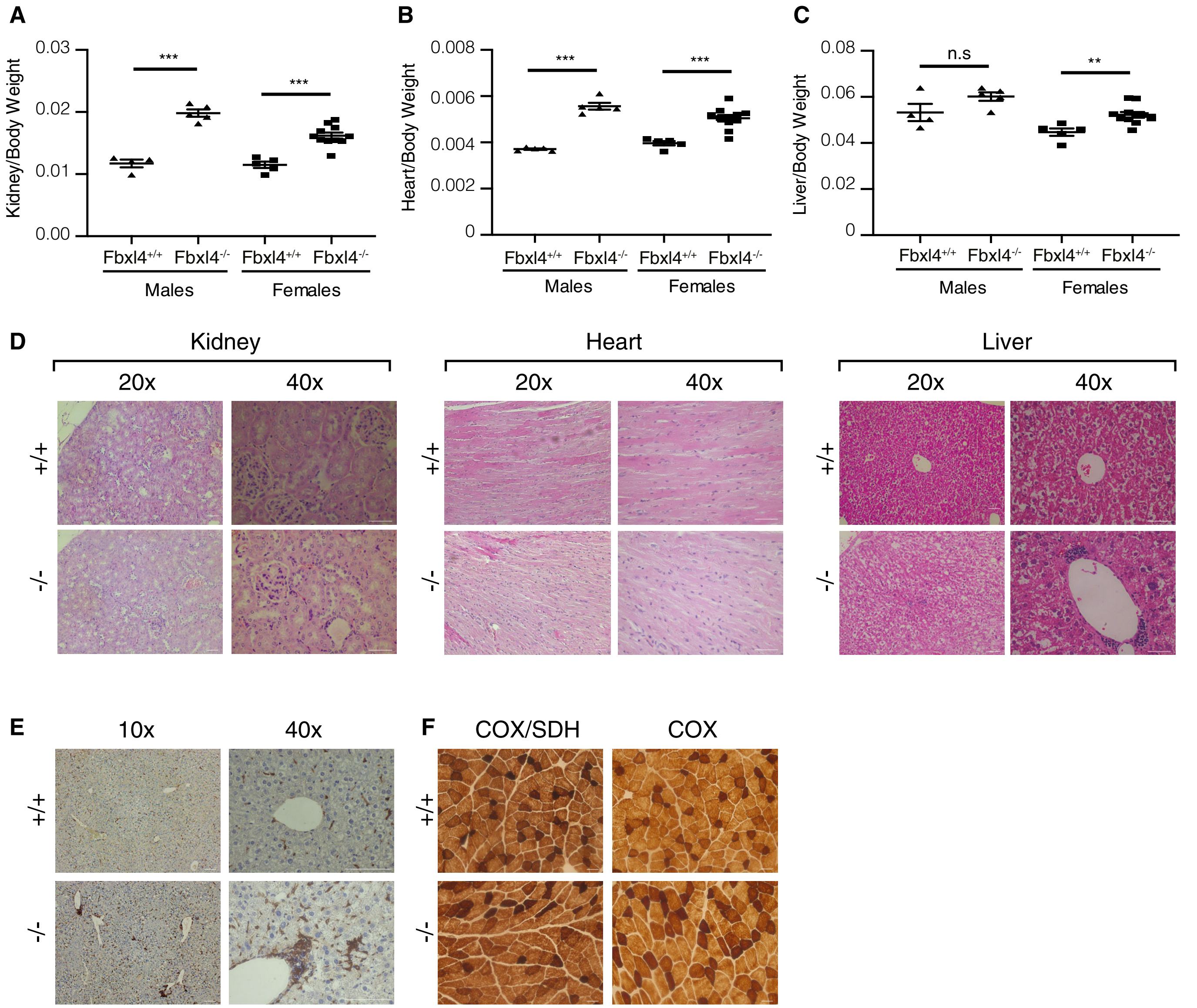

Figure EV1. Phenotypes of *Fbxl4* knockout mice.

A–C Organ-to-body weight ratios for kidney (A), heart (B), and liver (C) of 1-year-old animals. Data are presented as mean ± SEM. Student's *t*-test; n.s, $P > 0.05$; $^{**}P < 0.01$; $^{***}P < 0.001$.

D Representative images of H&E staining of kidney, heart, and liver tissue sections of $Fbxl4^{+/+}$ and $Fbxl4^{-/-}$ 1-year-old animals. Scale bar 50 μm.

E Representative images of Iba1 staining in liver tissue sections of $Fbxl4^{+/+}$ and $Fbxl4^{-/-}$ 1-year-old animals. Scale bar 100 μm.

F The individual COX and sequential COX/SDH reactions within quadriceps sections of $Fbxl4^{+/+}$ and $Fbxl4^{-/-}$ 1-year-old animals. Scale bar 50 μm.

© 2020 The Authors

Figure EV2. Protein steady-state levels in mitochondria of *Fbxl4* knockout animals.

A Label-free proteomic analysis of isolated liver mitochondria in wild-type versus *Fbxl4* knockout animals. Data are represented as $\log_{10}$ of the *P*-value on *y*-axis against $\log_2$ of knockout-to-control ratio ($\log_2$ fold change, logFC) on the *x*-axis.
B Western blot analysis of protein steady-state levels in isolated liver mitochondria from wild-type ($Fbxl4^{+/+}$) and Fbxl4 knockout ($Fbxl4^{-/-}$) animals.
C Label-free proteomic analysis of isolated kidney mitochondria in wild-type versus *Fbxl4* knockout animals. Data are represented as $\log_{10}$ of the *P*-value on *y*-axis against $\log_2$ of knockout-to-control ratio ($\log_2$ fold change, logFC) on the *x*-axis.
D Western blot analysis of protein steady-state levels in isolated kidney mitochondria from wild-type ($Fbxl4^{+/+}$) and Fbxl4 knockout ($Fbxl4^{-/-}$) animals.
E Label-free proteomic analysis of isolated heart mitochondria in wild-type versus *Fbxl4* knockout animals. Data are represented as $\log_{10}$ of the *P*-value on *y*-axis against $\log_2$ of knockout-to-control ratio ($\log_2$ fold change, logFC) on the *x*-axis.
F Western blot analysis of protein steady-state levels in isolated heart mitochondria from wild-type ($Fbxl4^{+/+}$) and Fbxl4 knockout ($Fbxl4^{-/-}$) animals.
G Venn diagram comparing the three mitoproteomic datasets generated by using an online tool accessible at http://bioinformatics.psb.ugent.be/webtools/Venn/.
H Blue native PAGE (BN–PAGE) analysis of respiratory chain complexes in wild-type ($Fbxl4^{+/+}$) and knockout ($Fbxl4^{-/-}$) animals.
I Relative respiratory chain enzymatic activities from liver mitochondria. Data are presented as mean ± SEM, $n = 5$. Student's *t*-test; $^{**}P < 0.01$.

© 2020 The Authors

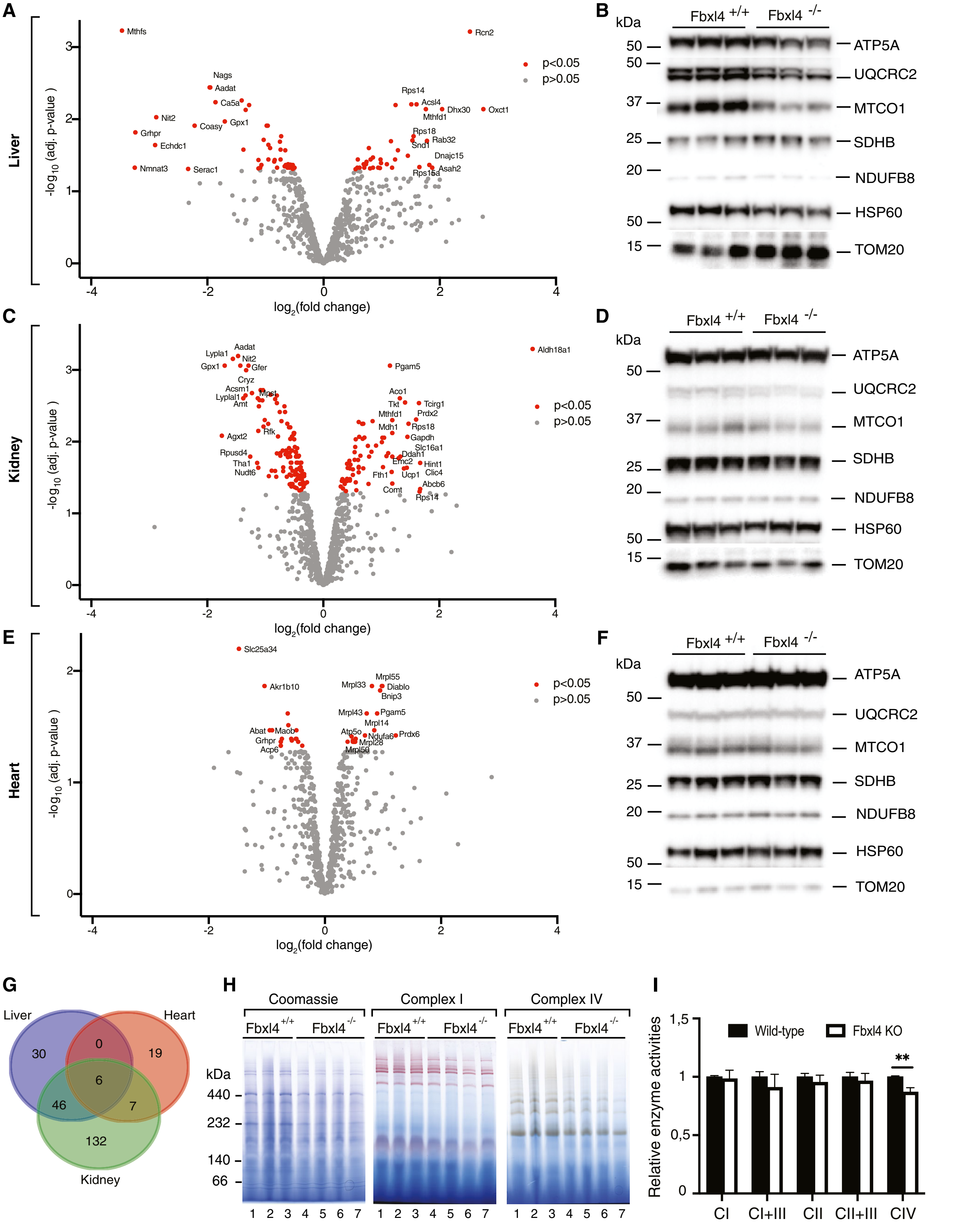
A
Liver
-log10 (adj. p-value)
log2(fold change)
p<0.05
p>0.05
Mthfs
Rcn2
Nags
Aadat
Ca5a
Nit2
Grhpr
Echdc1
Coasy
Gpx1
Nmnat3
Serac1
Rps14
Acsl4
Dhx30
Oxct1
Mthfd1
Rps18
Rab32
Snd1
Dnajc15
Rps15a
Asah2
B
kDa
Fbxl4 +/+
Fbxl4 -/-
ATP5A
UQCRC2
MTCO1
SDHB
NDUFB8
HSP60
TOM20
C
Kidney
Aldh18a1
Lypla1
Aadat
Nit2
Gpx1
Gfer
Pgam5
Cryz
Acsm1
Mpst
Lyplal1
Amt
Aco1
Tkt
Tcirg1
Mthfd1
Prdx2
Mdh1
Rps18
Rfk
Agxt2
Gapdh
Slc16a1
Rpusd4
Ddah1
Emc2
Tha1
Hint1
Nudt6
Fth1
Ucp1
Clic4
Comt
Abcb6
Rps14
D
E
Heart
Slc25a34
Mrpl55
Akr1b10
Mrpl33
Diablo
Bnip3
Mrpl43
Pgam5
Mrpl14
Abat
Maob
Atp5o
Ndufa6
Prdx6
Grhpr
Mrpl28
Acp6
Mrpl50
F
G
Liver
Heart
Kidney
30
0
19
6
46
7
132
H
Coomassie
Complex I
Complex IV
Fbxl4 +/+
Fbxl4 -/-
kDa
440
232
140
66
1 2 3 4 5 6 7
I
Relative enzyme activities
Wild-type
Fbxl4 KO
**
CI
CI+III
CII
CII+III
CIV

Figure EV2.

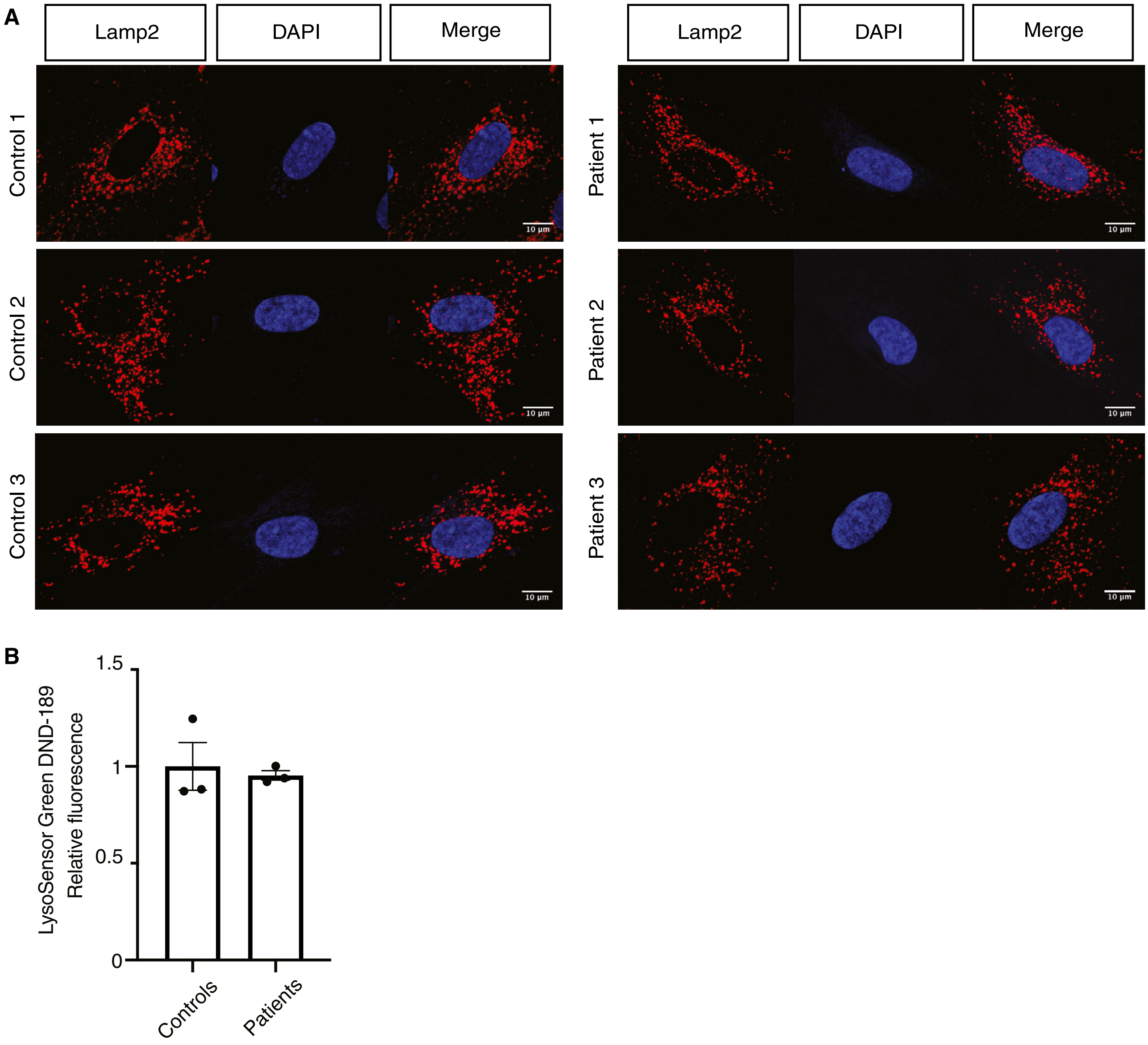

Figure EV3. Lysosomal network and pH measurement in *FBXL4* patient fibroblasts.

A Lamp2 immunofluorescence of control and patient fibroblasts. Scale bar, 10 µm.

B LysoSensor Green DND-189 fluorescence measurement. Data are presented as relative fluorescence value and represent mean ± SEM, $n = 3$.

Figure EV4. Autophagic flux in *FBXL4* patient fibroblasts.

A, B Steady-state protein levels in control and patient fibroblasts. Cells were grown in DMEM with 10% FBS (A) or in DMEM without FBS (B) for 3 h. After that, the cells were treated for 3 h with NH_4Cl to block autophagy, collected, and analyzed by Western blotting.

C, D Densitometric quantification of LC3-II (C) and p62 (D) normalized by GAPDH signal. Data are shown as mean ± SEM, $n = 4$.

E Western blot analysis of ubiquitin, p62, and LC3 accumulation in sucrose-purified mitochondria.

F Densitometric quantification ubiquitin, p62, and LC3-II. Signals from the control and FBXL4-mutated cells were considered as biological replicates, normalized to the average of controls, and displayed as mean ± SEM ($n = 3$ for each).

Source data are available online for this figure.

 © 2020 The Authors

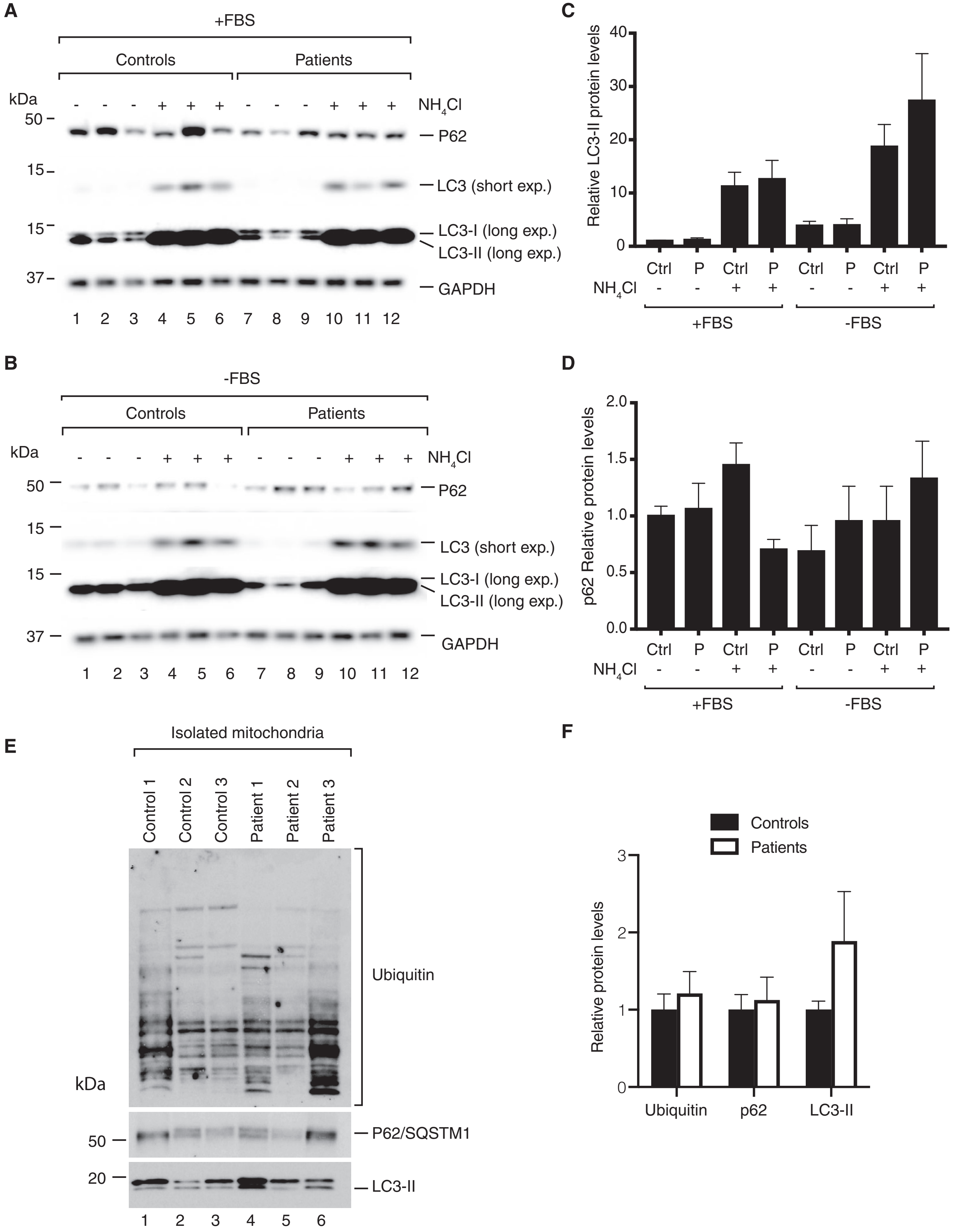
A
+FBS
Controls
Patients
kDa
NH4Cl
50
P62
15
LC3 (short exp.)
15
LC3-I (long exp.)
LC3-II (long exp.)
37
GAPDH
1 2 3 4 5 6 7 8 9 10 11 12
B
-FBS
Controls
Patients
kDa
NH4Cl
50
P62
15
LC3 (short exp.)
15
LC3-I (long exp.)
LC3-II (long exp.)
37
GAPDH
1 2 3 4 5 6 7 8 9 10 11 12
C
Relative LC3-II protein levels
Ctrl P Ctrl P Ctrl P Ctrl P
NH4Cl
+FBS
-FBS
D
p62 Relative protein levels
Ctrl P Ctrl P Ctrl P Ctrl P
NH4Cl
+FBS
-FBS
E
Isolated mitochondria
Control 1
Control 2
Control 3
Patient 1
Patient 2
Patient 3
Ubiquitin
kDa
50
P62/SQSTM1
20
LC3-II
1 2 3 4 5 6
F
Controls
Patients
Relative protein levels
Ubiquitin
p62
LC3-II

Figure EV4.
