# Supplementary figures and images for: FBXL4 deficiency increases mitochondrial removal by autophagy
Source: EMBO Mol Med. 2020 Jun 11;12(7):e11659. doi: 10.15252/emmm.201911659 (PMC7338799; doi:10.15252/emmm.201911659)

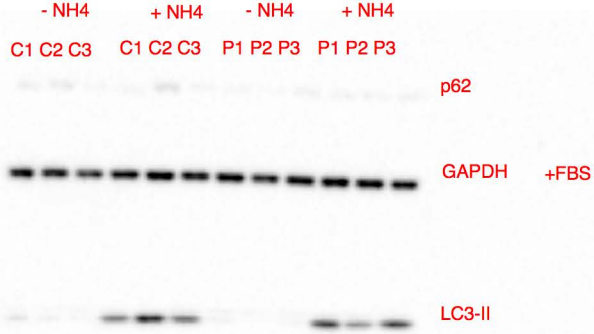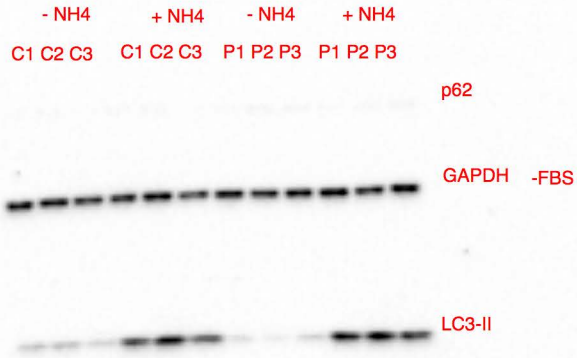

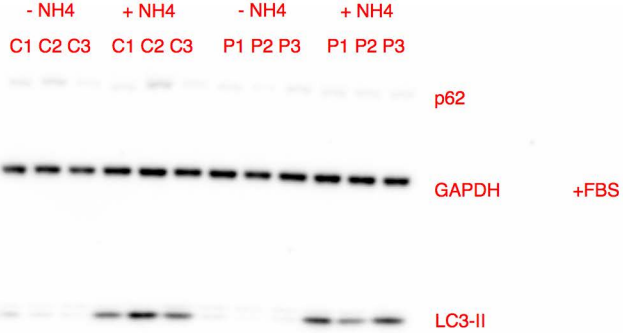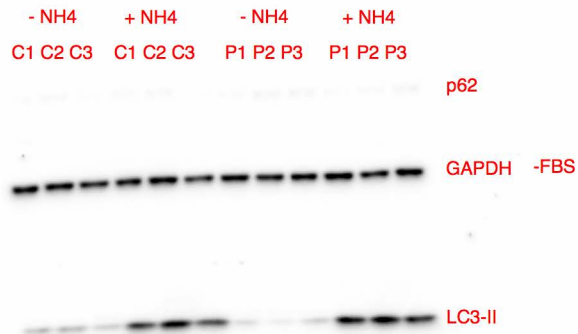

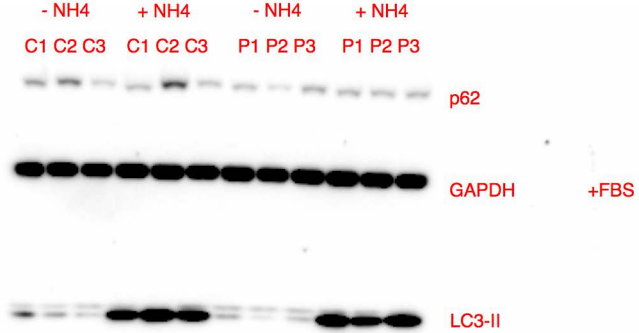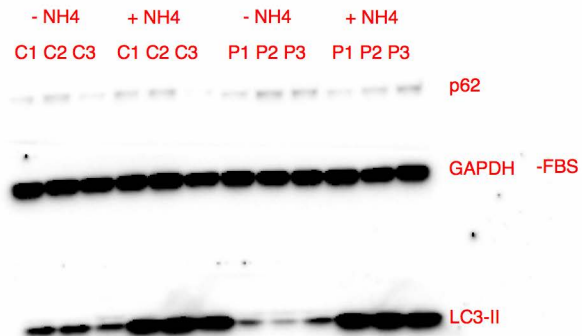

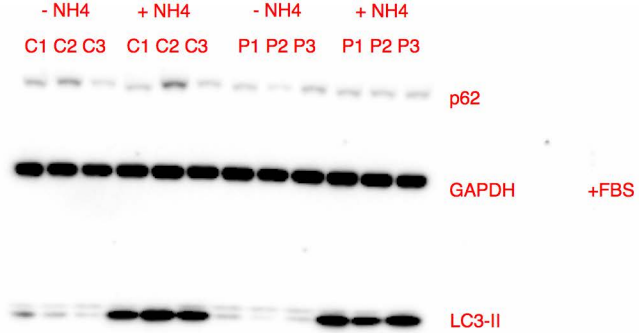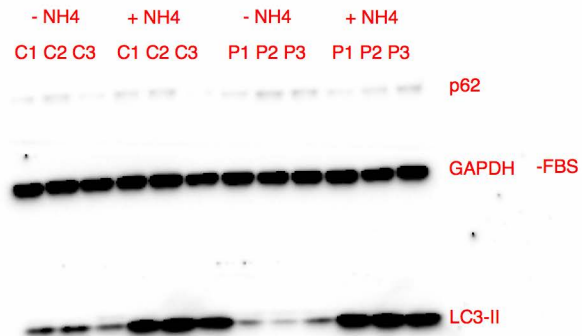

Mitox  
from fibro

C1 C2 C3 P1 P2 P3

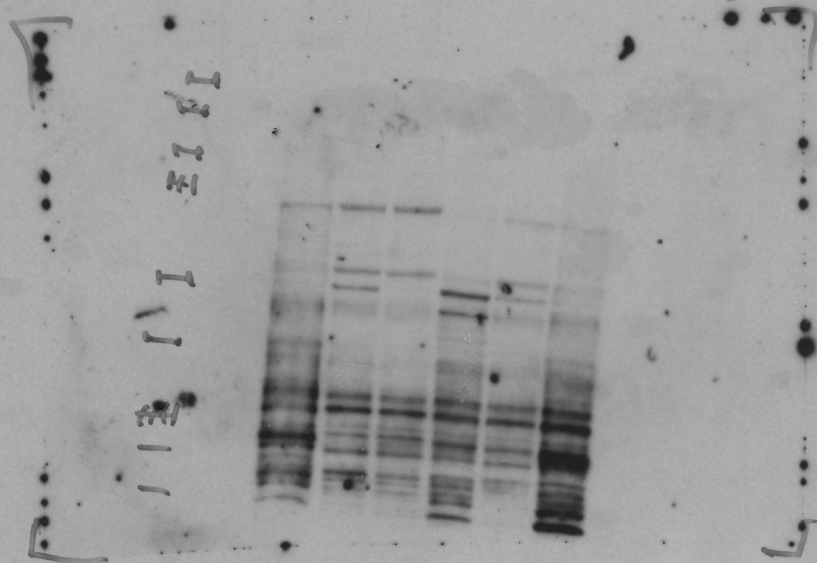

Poly-  
ubiquitin

10.03.20

Mitos

c1 c2 p1 p2 p3

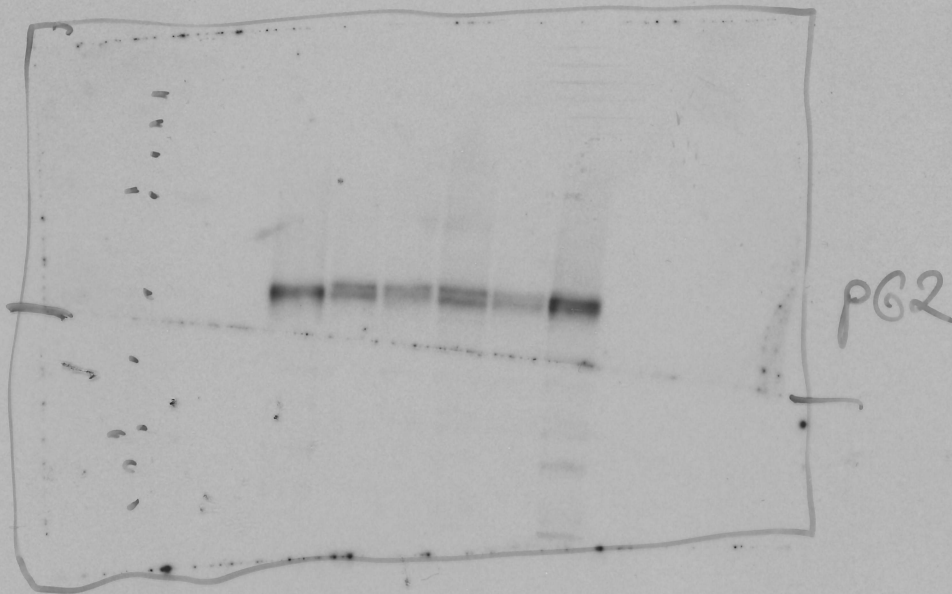

11.03.2020

mitos

c1 c2 c3 p1 p2 p3

37.  
25.  
20.  
15.

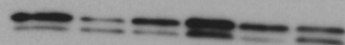

Le3

12.3.20

mitos

[ ..... ] V2Ae7  
c1c2e3p4p5

13.3.20

Supplement: Supplementary file 6 — Source Data for Expanded View [file EMMM-12-e11659-s006.pdf]
